# Exploring anatomical and geographical drivers of the microbiota in wild capybaras (*Hydrochoerus hydrochaeris*): Baseline Data for zoonotic risk assessment

**DOI:** 10.1371/journal.pone.0345409

**Published:** 2026-03-23

**Authors:** Milena Camargo, Jorge A. Marín-Sánchez, Plutarco Urbano, Nicolas Luna, Valentina Acevedo Ramírez, Julián A. Rodríguez, Davinzon Martínez, Carlos Porras, Luz H. Patiño, Marina Muñoz, Juan David Ramírez

**Affiliations:** 1 Centro de Investigaciones en Microbiología y Biotecnología–UR (CIMBIUR), School of Sciences and Engineering, Universidad del Rosario, Bogotá, Colombia; 2 Centro de Tecnología en Salud (CETESA), Innovaseq SAS, Funza, Cundinamarca, Colombia; 3 Grupo de Investigaciones Biológicas de la Orinoquia, Universidad Internacional del Trópico Americano (Unitrópico), Yopal, Colombia; 4 Instituto de Biotecnología -UN (IBUN), Universidad Nacional de Colombia, Bogotá, Colombia; 5 Center for Global Health and Inter-Disciplinary Research, USF Genomics Program, Department of Global, Environmental and Genomic Health Sciences, College of Public Health, University of South Florida, Tampa, Florida, United States of America; Universidade dos Açores Departamento de Biologia: Universidade dos Acores Departamento de Biologia, PORTUGAL

## Abstract

Capybaras (*Hydrochoerus hydrochaeris*) play important ecological, cultural, and economic roles in Colombia, particularly in the Orinoco region. Their adaptability to human-modified environments increases contact with humans and animals, raising concerns about their role in zoonotic pathogen transmission. However, their microbiota remains largely unexplored, limiting the understanding of potential health risks relevant to conservation and management. Bacterial communities were characterized in saliva, blood, and feces from 28 wild capybaras in Casanare, Colombia, through *16S-rRNA* amplicon sequencing on the Oxford Nanopore platform. Taxonomic profiling was performed with Kraken using the SILVA database. Microbial diversity was assessed in R (*phyloseq*, *vegan*), and differential abundance across sample types and sites was determined with ANCOM-BC2. After quality control, 196,281 reads were classified into 51 bacterial phyla and 1,779 genera. Proteobacteria, Firmicutes, and Bacteroidetes dominated the phylum-level profiles. At the genus level, composition varied by sample type and location. Fecal samples exhibited the highest bacterial richness and diversity, whereas blood samples displayed the lowest. Beta diversity analysis using Bray–Curtis dissimilarity and PCoA revealed distinct clustering by sample type, supported by PERMANOVA. Site-specific microbial signatures were identified: *Blautia* and *Prevotellaceae UCG-003* were enriched in Paz de Ariporo, while Trinidad showed higher genus-level diversity. Saliva samples displayed the strongest contrasts, with *Acinetobacter* and *Thiobacillus* enriched in Trinidad, and *Tyzzerella*, *Arthrobacter*, and *Histophilus* more abundant in Paz de Ariporo. Core microbiota analysis revealed the fewest core genera in blood (one genus), a moderate overlap in saliva (nine genera), and the highest number in feces (13 genera). Distinct patterns in bacterial composition and diversity across sample types and locations were demonstrated in wild capybaras. Both anatomical site and geographic origin influenced the microbiota. These findings provide baseline data to support future research on wildlife microbiota and its potential role in ecosystem health, conservation, and zoonotic transmission.

## Introduction

Capybaras (*Hydrochoerus hydrochaeris*), the largest rodents in the world, are widely distributed across South America. As herbivorous and semi-aquatic mammals, they depend on lakes and rivers, displaying a remarkable ability to adapt to human-modified environments such as agricultural and urban areas [1]. Ecologically, they play a vital role in wetland and savanna ecosystems through their grazing behavior, which contributes to nutrient cycling and the redistribution of organic matter, influencing plant communities and supporting other species [2,3].

Beyond their ecological role, capybaras hold economic importance in several South American countries, including Colombia, where they are used mainly for meat and leather [4,5]. Although they are culturally familiar and widely accepted, wild populations face pressures in some regions due to illegal hunting. In Arauca, a department located in northeastern Colombia along the border with Venezuela, poaching driven by cross-border trade has impacted local populations. In contrast, Casanare situated in the eastern plains (Llanos Orientales) of Colombia shows marked differences among municipalities: while areas such as Paz de Ariporo have made efforts to protect habitats and control poaching, municipalities like Hato Corozal still experience illegal exploitation. Efforts by rancher associations to promote sustainable use are challenged by limited technical capacity and organizational weaknesses. Population densities in Colombia range from 0.1 to 7.1 individuals per hectare, highlighting the need for regionally adapted management plans [4,5].

In recent decades, capybaras have expanded considerably in human-altered landscapes due to increased food availability, fewer natural predators, high reproductive rates, and, in some areas, even domestication or use as pets, showing remarkable adaptability [6]. Along with this expansion, increasing attention has been given to the diverse roles of their microbial communities, which may include both beneficial and pathogenic members. For example, capybaras are involved in the epidemiology of *Rickettsia rickettsii*, the causative agent of Brazilian spotted fever, serving as hosts for *Amblyomma sculptum* ticks and amplifying infection cycles [7]. However, this expansion raises ecological and public health concerns, particularly regarding zoonotic disease transmission.

Capybaras harbor diverse microbial communities that may include parasites, viruses, and bacteria. Gastrointestinal parasites such as *Neospora caninum*, endoparasites and Intestinal helminths have been reported in wild populations in South America [8–10]. Viral detections have included mimiviruses, vaccinia virus (VACV), rabies virus, and even a coronavirus identified during a diarrheal outbreak, suggesting a potential role in the ecology of emerging infections, particularly in peri-urban areas. Nevertheless, bacterial communities remain the most studied, as they provide valuable insights into host physiology, health, and the zoonotic potential of these rodents [11,12].

Despite their ecological relevance, commercial value, and increasing interactions with humans and livestock, studies on the microbiota of capybaras remain scarce, particularly in Colombia. A deeper understanding of their microbial communities is essential not only to evaluate their potential role as reservoirs or amplifiers of pathogens, but also to identify beneficial symbionts that may influence host health. Strengthening this knowledge will provide a framework to assess health risks, support conservation efforts, and guide the development of regionally adapted management strategies [13,14].

Advances in next-generation sequencing (NGS) technologies have revolutionized microbial ecology by enabling the identification and quantification of bacterial communities without the need for cultivation. This is particularly relevant in wildlife research, where many host-associated microorganisms are not easily cultured under standard laboratory conditions [15], In capybaras, 16S rRNA gene sequencing serves as a powerful tool for taxonomic profiling of their microbiota, providing a molecular fingerprint to explore host–microbe interactions. More recently, third-generation sequencing platforms such as Oxford Nanopore have enabled real-time long-read sequencing, enhanced taxonomic resolution and offered functional and ecological insights into microbial communities. These approaches are especially valuable for elucidating the role of microbiota in capybara physiology, health, and their potential as reservoirs of zoonotic pathogens, particularly in anatomically and functionally complex sites [16].

In this context, the objective of this study is to characterize the bacterial communities present in paired samples of saliva, blood, and feces from capybaras in Paz de Ariporo and Trinidad, Casanare, Colombia. Using ONT 16S rRNA sequencing, we aim to generate high-resolution taxonomic profiles and explore microbial diversity across anatomical niches, contributing novel data on a species of ecological and economic importance that remains understudied in microbiome research. Additionally, this study provides baseline information on microorganisms with potential pathogenic relevance, supporting future assessments of their implications for wildlife health, conservation, and zoonotic risk.

## Materials and methods

### Ethical statement

This study was approved and supervised by Universidad del Rosario’s School of Medicine and Health Sciences, Research Ethics Committee (Colombia: resolution CEI-DVO005 1585-CV1427). The Colombian National Environmental Licensing Authority Resolution 01877 October 22nd, 2018, permitted the collection of specimens of wild species having biological diversity for non-commercial scientific research purposes.

### Capybara sample collection

**Fig 1:**
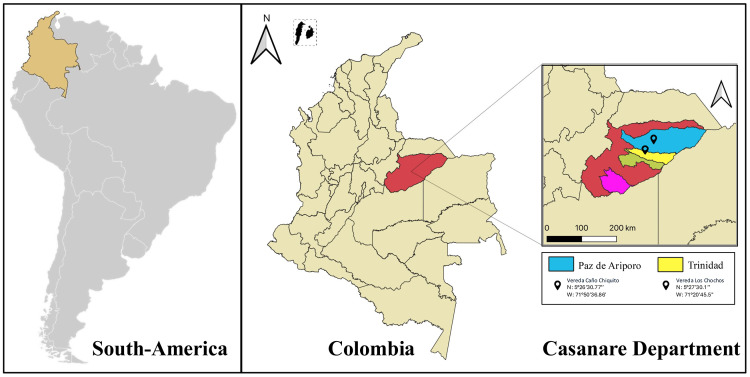
Geographical locations of the municipalities of Paz de Ariporo and Trinidad, Casanare, Colombia, where Capybara samples were collected.

Between March and April 2023, capybaras were captured using the horseback lassoing method, which consisted of lassoing the animal by the neck and tying its four limbs once it lost balance during the chase [17], in two municipalities (Paz de Ariporo and Trinidad) in the department of Casanare, located in the Eastern Plains region of Colombia (Fig 1). Captured individuals were anesthetized with ketamine. Blood samples (0.5 mL) were collected from the femoral vein using 5 mL syringes. Oral secretions were obtained by oropharyngeal swabbing at the level of the snout, and fecal samples were collected directly from the animals during handling when possible, or by rectal swabbing. Buccopharyngeal and fecal samples were stored and transported in Eppendorf tubes containing RNAlater and preserved at 4 °C for subsequent analyses. After sample collection, all individuals were released upon recovery from anesthesia.

### Nucleic acid isolation from Capybara samples

Genomic DNA was extracted from oral swabs, blood, and fecal samples obtained from wild capybaras. For oral swab samples, the Quick Extract™ DNA Solution (Lucigen®) was used, whereas blood samples were processed with the DNA Isolation Kit HighPure PCR Template Preparation (Roche® Life Science). Fecal samples, obtained via rectal swabbing, were homogenized by disruption with ceramic beads for 5 min at 30 Hz using a TissueLyser II disruptor (Qiagen) and extracted using the Stool DNA Isolation Kit (Norgen®, Biotek Corp.). All extractions were carried out following the manufacturers’ protocols.

The concentration and purity of the extracted DNA were assessed using a NanoDrop 2000/2000c spectrophotometer (Thermo Fisher Scientific), based on absorbance at 260/280 nm. Negative extraction controls (molecular-grade water) were included throughout the procedure to monitor potential cross-contamination. All samples were stored at −20 °C until further processing.

### Amplification of the 16S rRNA gene and sequencing of samples using Oxford Nanopore Technologies (ONT)

To assess bacterial community composition, a conventional PCR targeting the full-length 16S-rRNA gene was performed using universal primers 27F (5’-AGAGTTTGATCCTGGCTCAG-3’) and 1492R (5’-GGTTACCTTGTTACGACTT-3’), which allowed for high-resolution taxonomic identification of bacterial communities [18,19]. PCR reactions were carried out in a final volume of 13 μL, containing LongAmp® Taq 2X Master Mix (New England Biolabs) at a final concentration of 1X, 1 μM of each primer, 2.25 μL of nuclease-free water, and 2 μL of genomic DNA template.

The thermal cycling protocol consisted of an initial denaturation at 94 °C for 30 seconds, followed by 30 cycles of denaturation at 94 °C for 30 seconds, annealing at 47.9 °C for 1 minute, and extension at 65 °C for 1 minute and 15 seconds. A final extension step was performed at 65 °C for 10 minutes. A positive control consisting of DNA from a known bacterial isolate was included, as well as a no-template control (PCR-grade water).

PCR products were visualized on 1.2% agarose gels using 3 μL of Gel Loading Dye, Blue (6X) and 3 μL of the amplified product. A 1 kb DNA Ladder (New England Biolabs) was used as the molecular weight marker.

For long-read sequencing, barcoding was performed using the EXP-NBD196 kit (Oxford Nanopore Technologies). PCR products were purified using AMPure® XP beads (Beckman Coulter). Library preparation and adapter ligation followed the SQK-LSK110 protocol (Oxford Nanopore Technologies). The final library was loaded onto an R9.4 flow cell (FLO-MIN106; Oxford Nanopore Technologies), and sequencing was performed on the MinION™ Mk1C platform using the MinKNOW software.

### Sequencing data processing and taxonomic profiling

For basecalling, the pod5 files generated by the sequencing were processed using Dorado version 0.7.2, applying the super-accurate model, which considered reads with a Qscore equal or greater than 10. The average Phred quality score was evaluated using the NanoPack tool [20]. For taxonomic assignment, the SILVA v132.16s database [21] was used, which is widely employed in microbial community studies due to its high coverage and manual curation. Read clustering and taxonomic profiling were performed using Kraken [22]. Subsequently, the results generated by Kraken were visualized using Pavian [23].

Bacterial community composition was analyzed using tools within the R environment v4.4.1. The analysis integrated the packages phyloseq and ggplot2 [24,25]. Rarefaction curves were generated to assess the diversity of ASVs in each sample based on the number of sequencing reads, ensuring adequate coverage before downstream analyses. Subsequently, taxonomic counts were normalized and transformed into relative abundances. Stacked bar plots were generated to visualize the taxonomic profile.

### Bacterial alpha and beta diversity estimation

Alpha diversity indices were calculated using the estimate_richness function from the phyloseq package, focusing on four metrics: Observed (species richness), Shannon (richness and evenness), Inverse Simpson (dominance and evenness), and Chao1 (estimated richness including rare taxa). These indices were used to compare alpha diversity between sample types (feces, saliva, and blood) and the two sampling locations (Paz de Ariporo and Trinidad). The data were reshaped into long format using the pivot_longer function from the tidyr package to facilitate visualization, which was performed using ggplot2.

Prior to group comparisons, the Shapiro-Wilk test was applied to assess the normality of each diversity index. Depending on data distribution and the number of groups, either parametric tests (Student’s t-test or ANOVA) or non-parametric tests (Wilcoxon or Kruskal-Wallis) were used to evaluate statistically significant differences.

Beta diversity was assessed using the Bray-Curtis dissimilarity index, after transforming the data into relative proportions. Non-metric multidimensional scaling (NMDS) and Principal Coordinates Analysis (PCoA) were then performed, and results were visualized using the plot_ordination function from the phyloseq package. Differences in microbial community structure between groups were evaluated through permutational multivariate analysis of variance (PERMANOVA) using the adonis2 function from the vegan package, with 9,999 permutations [24,26]. Homogeneity of group dispersions (PERMDISP) was assessed with betadisper, followed by a permutation test using permutest. Additionally, analysis of similarity (ANOSIM) was conducted to further evaluate differences in microbial community composition according to sample type and sampling location.

### Differential abundance analysis

Differential abundance analysis was performed using the ANCOM-BC2 package (ANCOMBC R package v2.4.0) to assess variations in bacterial genera across groups, including sample types and sampling locations. Quality filtering steps included the removal of low-abundance features (library size cutoff of 1000), adjustment for structural zeros, and multiple testing correction using the Holm method [27]. To enable pairwise comparisons, the reference category was alternated among sample types (e.g., blood, feces), allowing for the estimation of log2 fold changes in genus-level abundance. The W-statistic was used to quantify the strength of evidence for differential abundance. Finally, core bacterial genera were identified by applying a prevalence-based threshold, considering taxa present in at least 70% of samples within each biological source (saliva, blood, and feces) [28].

## Results

### Overview of *16S-rRNA* sequencing output and bacterial community profiles

A total of 28 capybaras were sampled, with 14 individuals from Paz de Ariporo and 14 from Trinidad, both located in the department of Casanare (Colombia). For each animal, three biological sources were sampled: saliva, feces, and blood, resulting in a total of 84 samples analyzed.

Following 16S-rRNA long-amplicon-based sequencing and subsequent quality control through trimming and filtering, a total of 196,281 high-quality reads were retained. These corresponded to 64,956 reads from saliva, 57,440 from feces, and 73,885 from blood. Taxonomic classification assigned the sequences to 51 bacterial phyla and 1,786 bacterial genera. Among the three sample types, feces exhibited the highest bacterial taxa, with 1,284 identified genera, followed by saliva (1,228 genera) and blood (907 genera) (S1 Table). Proteobacteria was the most abundant phylum across all sample types, representing between 37.5% and 52.5% of the relative abundance, followed by Firmicutes, which accounted for 23.4% to 35.4% and Bacteroidetes (range 5.0% to 17.2%).

At the genus level, the most dominant taxa varied by sample type. In saliva, *Bacillus* (9.5%) and *Moraxella* (7.5%) were the most abundant. Fecal samples were dominated by *Pseudohongiella* (13.5%) and *Bacillus* (12.4%). In blood samples, *Bacillus* reached a relative abundance of 36.3%, followed by *Mesorhizobium* (16.9%).

### Taxonomic profiles and diversity in feces, saliva, and blood from Paz de Ariporo

**Fig 2:**
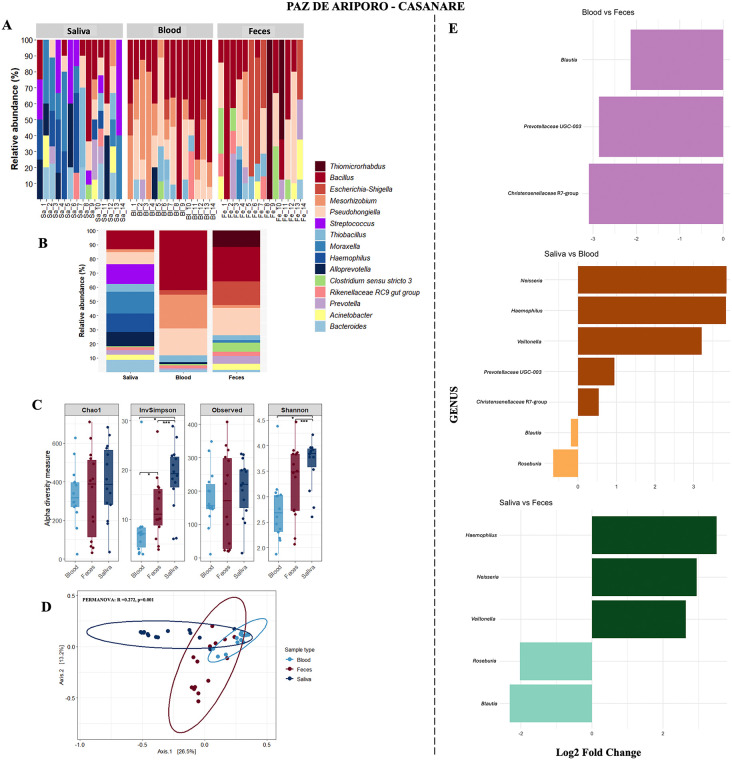
Microbial community composition and diversity in fecal, salivary, and blood samples from capybaras in Paz de Ariporo, Casanare. **(A)** Relative abundance of the 15 most frequent bacterial genera per individual. **(B)** Average relative abundance of these 15 genera grouped by sample type. **(C)** Alpha diversity indices (Observed richness, Chao1, Shannon, Simpson) across sample types. **(D)** Beta diversity analysis (PCoA based on Bray–Curtis dissimilarity) showing clustering by sample type. **(E)** Differentially abundant genera across sample types identified using ANCOM-BC analysis. Significance codes: *p < 0.05; **p < 0.01; ***p < 0.001. All visualizations were generated in RStudio using the ggplot2 package.

In Paz de Ariporo, microbial community composition at the genus level differed markedly among feces, saliva, and blood. Fecal samples were dominated by *Pseudohongiella*, *Bacillus*, and *Escherichia-Shigella*, while saliva samples showed a higher relative abundance of *Moraxella*, *Streptococcus*, and *Haemophilus*. Blood samples, in contrast, were primarily characterized by *Bacillus* and *Mesorhizobium* (Fig 2A and 2B).

Alpha diversity comparisons revealed significant differences across sample types. Saliva exhibited the highest diversity, while blood had the lowest values. Specifically, the Shannon index showed saliva to be significantly more diverse than blood and feces (Kruskal Wallis, p = 0.00244; Dunn’s post hoc with FDR correction: Saliva vs Blood, p = 0.0484; Saliva vs Feces, p = 0.0008). Similar patterns were observed with the Simpson index (Kruskal Wallis, p = 0.000072; Dunn’s post hoc: Saliva vs Feces, p = 0.0002; Feces vs Blood, p = 0.0334; Saliva vs Blood, p = 0.0365) (Fig 2C).

Beta diversity analysis based on Bray–Curtis dissimilarity revealed clear clustering of microbial communities by sample type, with feces, saliva, and blood forming distinct groups (Fig 2D). This separation was confirmed by PERMANOVA, which indicated statistically significant differences among sample types.

The ANCOM-BC analysis identified several differentially abundant genera across sample sources. *Blautia* was consistently significant across comparisons, while *Prevotellaceae UCG-003*, *Christensenellaceae R-7 group*, *Neisseria*, *Haemophilus*, *Veillonella*, and *Roseburia* also showed sample-specific enrichment (Fig 2E).

### Taxonomic profiles and diversity in feces, saliva, and blood from Trinidad

**Fig 3:**
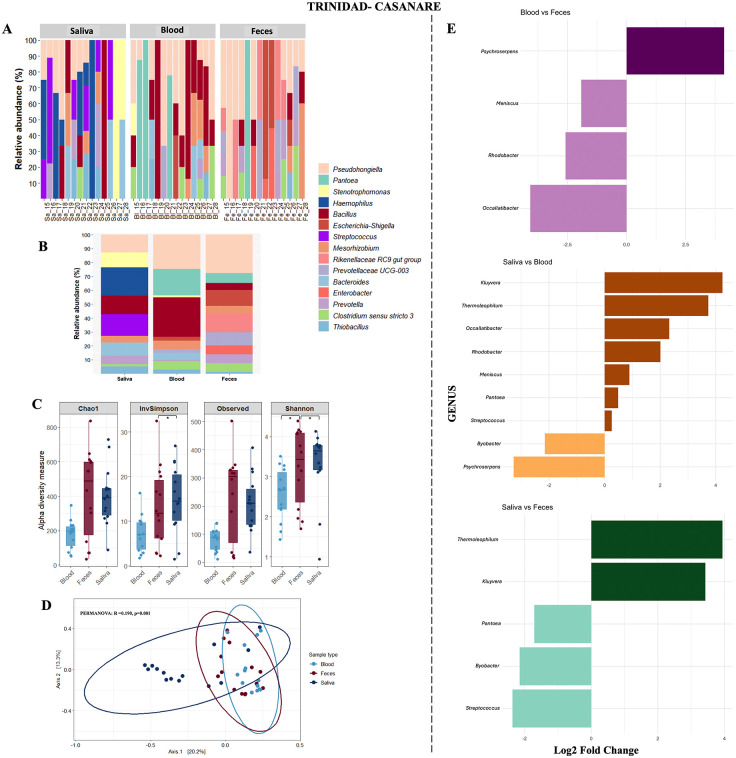
Microbial community composition and diversity in fecal, salivary, and blood samples from capybaras in Trinidad, Casanare. **(A)** Relative abundance of the 15 most frequent bacterial genera per individual. **(B)** Average relative abundance of these 15 genera grouped by sample type. **(C)** Alpha diversity indices (Observed richness, Chao1, Shannon, Simpson) across sample types. **(D)** Beta diversity analysis (PCoA based on Bray–Curtis dissimilarity) showing clustering by sample type. **(E)** Differentially abundant genera across sample types identified using ANCOM-BC analysis. Significance codes: *p < 0.05; **p < 0.01; ***p < 0.001. All visualizations were generated in RStudio using the ggplot2 package.

In Trinidad, fecal samples were dominated by *Pseudohongiella* and *Escherichia-Shigella*, while saliva was enriched in *Stenotrophomonas* and *Streptococcus*. Blood samples at this site were mainly characterized by *Bacillus* and *Pantoea* (Fig 3A and 3B).

Diversity comparisons also revealed significant differences. According to the Shannon index, feces were more diverse than blood, while saliva was more diverse than feces (Kruskal Wallis, p = 0.0240; Dunn’s post hoc: Feces vs Blood, p = 0.0184; Saliva vs Feces, p = 0.0206). Differences were also detected with the Simpson index, where saliva was significantly more diverse than feces (Kruskal Wallis, p = 0.0235; Dunn’s post hoc: Saliva vs Feces, p = 0.0121) (Fig 3C). Beta diversity analyses revealed consistent clustering of samples by type, with feces, saliva, and blood forming distinct communities. These patterns were statistically supported by PERMANOVA (Fig 3D). ANCOM-BC analysis indicated a broader range of differentially abundant genera compared to Paz de Ariporo. Enriched taxa included *Psychroserpens*, *Meniscus*, *Rhodobacter*, *Kluyvera*, *Thermoleophilum*, *Pantoea*, *Occallatiobacter*, *Streptococcus*, and *Bryobacter* (Fig 3E).

### Comparative microbial profiles of saliva, blood, and feces between sampling sites

**Fig 4:**
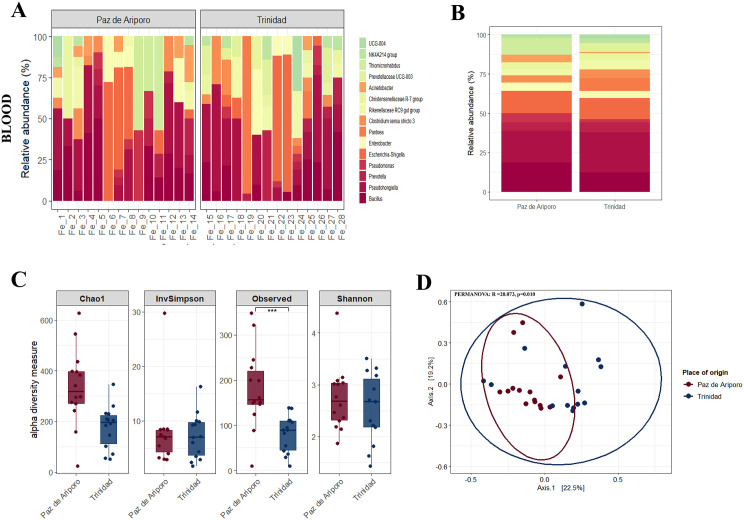
Bacterial community composition and diversity in blood samples of capybaras. Panel A shows the relative abundance of the 15 most frequent bacterial genera across all samples, while panel B presents the average abundance of these genera grouped by sampling location. Panel C displays alpha diversity indices (Observed, Shannon, Inverse Simpson, and Chao1), and panel D shows beta diversity metrics across sites. Significance codes: *p < 0.05; **p < 0.01; ***p < 0.001. All visualizations were generated in RStudio using the ggplot2 package.

Unlike the previous sections, which described microbial composition within each municipality separately (Paz de Ariporo and Trinidad), this analysis directly compared the same sample types (saliva, blood, and feces) across the two locations to identify geographic differences. The bacterial composition revealed site-specific patterns (Fig 4 and S1 Fig). In saliva samples (S1A Fig), *Bacillus*, *Neisseria*, and *Veillonella* were more abundant in individuals from Trinidad, whereas *Haemophilus* was more prominent in samples from Paz de Ariporo. In blood samples (Fig 4A and 4B), capybaras from Paz de Ariporo exhibited higher levels of *Bacillus* and *Mesorhizobium*, while *Pseudohongiella* was more abundant in individuals from Trinidad. Blood was the sample type with the lowest bacterial diversity, in contrast to fecal samples, which exhibited the highest overall diversity. In feces (S1B Fig), the taxonomic composition was largely similar between both locations, with the exception of an increased abundance of *Prevotellaceae* in samples from Paz de Ariporo.

Regarding diversity indices, statistically significant differences were observed only in blood samples, with the observed richness index showing significance (Kruskal Wallis test, p = 0.000617) (Fig 4C). Similarly, beta diversity analysis revealed significant differences in blood samples (Fig 4D), whereas no clear clustering patterns were detected for saliva or fecal samples (S1C and D Fig). Additionally, the ANCOM-BC analysis identified differentially abundant genera exclusively in saliva samples between locations: *Acinetobacter*, *Thiobacillus*, and *Clostridium sensu stricto 3* were significantly more abundant in Trinidad, while *Tyzzerella*, *Arthrobacter*, and *Histophilus* were enriched in Paz de Ariporo (S2 Fig).

### Core microbiota and taxonomic overlap across biological sources

**Fig 5:**
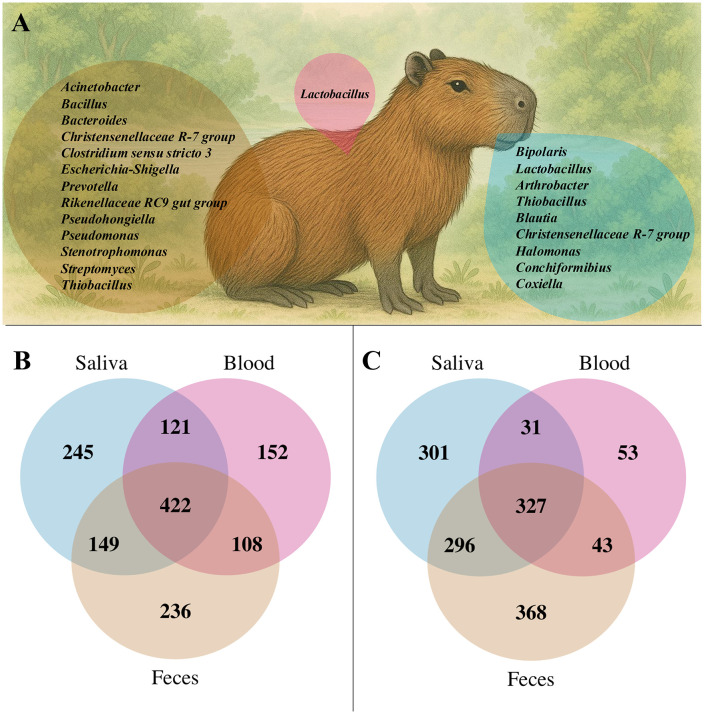
Core microbiota and distribution of bacterial genera across biological sources. **(A)** Core bacterial genera identified in at least 70% of samples for each biological source (saliva, blood, and feces). Venn diagrams showing the distribution of bacterial genera shared among and unique to saliva, blood, and fecal samples from capybaras. **(B)** Samples from Paz de Ariporo. **(C)** Samples from Trinidad.

After assessing taxonomic composition and differentially abundant genera across sample types and locations, we next sought to identify bacterial groups consistently present across individuals. To this end, we defined a core microbiome as genera detected in at least 70% of the samples, a criterion widely applied in microbiome studies [28]. This approach highlights persistent members of the community and their overlap across biological sources, offering insights into microbial components potentially linked to host physiology and stability. The results showed that blood had the fewest core genera, with only one genus consistently detected across samples. In contrast, saliva displayed a moderate overlap with nine core genera, while feces exhibited the highest number, with 13 core genera consistently present (Fig 5A).

In addition, to assess taxonomic overlap across sample types, we generated Venn diagrams comparing bacterial genera detected in saliva, blood, and feces by sampling location (Paz de Ariporo and Trinidad). This site-specific analysis revealed that samples from Paz de Ariporo exhibited greater taxonomic overlap across sample types compared to those from Trinidad (Fig 5B and 5C, S2 Table). In both locations, however, saliva and feces consistently shared more genera with each other than did with blood, a pattern particularly pronounced in Trinidad.

## Discussion

In Colombia, the commercial hunting of capybaras is permitted for human consumption, highlighting the need to better understand the ecology and microbiota of this species due to its ecological relevance and increasing economic importance [29]. Microbiome profiling of wild-caught individuals provides baseline information on microbial composition and diversity, which may contribute to future studies evaluating host health, environmental interactions, and potential zoonotic interfaces. Such information supports wildlife health surveillance and sustainable management strategies within conservation and public health.

The analysis revealed that Proteobacteria, Firmicutes, and Bacteroidetes were the most dominant phyla, consistent with previous reports in capybaras from Brazil and Venezuela [30,31]. Variations in relative abundance are consistent with environmental and dietary influences previously described in capybara populations. These patterns highlight the dynamic nature of microbiomes in response to local ecological conditions [30,31].

Marked differences in microbial community structure were observed between sample types from the same individuals (Figs 2 and 3), including variation in taxonomic composition, diversity indices, and differential abundance patterns. These findings are consistent with the distinct ecological niches and physiological conditions of each anatomical site [30,32–35]. Fecal samples showed enrichment of taxa commonly associated with polysaccharide degradation, including *Prevotella*, *Clostridium sensu stricto*, and *Christensenellaceae* R-7 group [36]. Similar site-specific microbial differentiation has been described in other rodent species, where local physicochemical conditions shape microbial composition and function [37,38].

The oral cavity also displayed distinctive microbial profiles (Figs 2 and 3). Capybaras possess well-developed salivary glands that secrete enzyme-rich saliva, including alpha-amylase, mucins, lysozyme, and IgA [32,39]. These secretions are known to contribute to microbial regulation and digestive processes. *Streptococcus* was identified as differentially abundant in salivary samples, which may be associated with salivary physiological conditions that influence oral microbial composition. Similar associations between salivary factors and oral microbiota structure have been reported in other mammals [40], although further studies in capybaras are required to confirm these relationships.

At the intestinal level, predominant bacterial genera included *Bacillus*, *Bacteroides*, *Blautia*, *Roseburia*, *Clostridium sensu stricto*, *Prevotella*, *Streptomyces*, and *Lactobacillus* (Figs 2 and 3). These genera are commonly associated with plant polysaccharide fermentation and nitrogen metabolism, consistent with the herbivorous diet of capybaras [41,42]. The taxonomic profiles observed in this study are consistent with previous reports of gut microbiota composition in capybaras and other rodents, particularly regarding the presence of taxa associated with plant polysaccharide degradation. Previous metagenomic studies in capybaras have reported a high abundance of genes encoding carbohydrate-active enzymes (CAZymes), mainly associated with Fibrobacteres and Bacteroidetes, which are involved in the degradation of cellulose and hemicellulose [30,43]. These findings support the relevance of these microbial groups in the digestive ecology of this species. Although functional activity was not directly assessed in this study, the taxonomic composition observed is consistent with previously reported functional potential in capybaras and other herbivorous rodents, supporting the role of gut microbiota in fiber digestion in this species.

Differences in microbiota composition were observed between sampling sites, with several genera showing differential abundance (Fig 4 and Figs S1 and S2). Because environmental and host-related variables were not recorded in this study, the drivers of these differences cannot be determined. Previous studies in mammals have shown that factors such as diet, habitat, and host characteristics can influence gut microbiota composition [38,44]; however, further studies specifically designed to evaluate these variables are needed in wild capybaras.

*Lactobacillus* was identified as part of the core microbiome in both fecal and blood samples (Fig 5). Members of this genus are widely reported in mammalian gut microbiota and are commonly associated with host metabolic and immune functions [45,46]. However, the detection of *Lactobacillus* DNA in blood samples should be interpreted with caution, as blood represents a low-biomass environment and detection does not necessarily indicate viable bacteria. Further studies are required to determine whether this finding reflects biological processes or circulating bacterial DNA in wild capybaras.

The blood microbiota remains poorly characterized, particularly in wild mammals such as capybaras. In other animals, bacterial DNA has been detected in blood and associated with different physiological and pathological states [47–50]. In humans, variations in blood microbiota composition have been associated with metabolic and cardiovascular conditions [51]. In the present study, blood samples showed relative abundances of Proteobacteria, Firmicutes, and Bacteroidetes, which are also commonly found in the gut microbiota. This pattern is consistent with potential microbial exchange between compartments; however, further studies are required to confirm biological relevance (Figs 2 and 3).

Given that blood represents a low-biomass sample, the risk of background contamination from reagents, laboratory environment, or sample handling must be considered. In this study, negative controls were included during 16S rRNA PCR amplification and sequencing to monitor potential contamination and minimize technical bias. Nevertheless, environmental-associated genera such as *Bacillus*, *Mesorhizobium*, and *Pantoea* are commonly found in soil, water, and plant-associated environments, and their detection in blood samples may reflect environmental or reagent-derived DNA rather than true biological presence. Therefore, these findings should be interpreted with caution. These limitations are inherent to low-biomass microbiome studies and highlight the need for complementary approaches to better define the biological relevance of these taxa in wildlife blood microbiota [52,53].

Capybaras have previously been reported as carriers of several zoonotic pathogens, including *Leptospira*, *Rickettsia rickettsii*, *Anaplasma*, *Ehrlichia*, and *Salmonella* [7,54–56]. In this study, bacterial genera such as *Escherichia-Shigella*, *Pseudomonas*, *Stenotrophomonas*, and *Coxiella* were detected, all of which include species associated with human infections. These findings highlight the importance of monitoring microbial diversity in wildlife populations, particularly in regions where capybaras interact with livestock and human populations [57].

The detection of *Pseudomonas* and *Stenotrophomonas* is noteworthy (Fig 5). Although many species within these genera are environmental and non-pathogenic, both include members recognized as opportunistic pathogens, particularly in immunocompromised individuals, and some have been increasingly associated with multidrug resistance [58,59]. These bacteria have also been reported in ticks and other ectoparasites [60,61]. However, their presence does not demonstrate vector competence, and their ecological or epidemiological role remains nuclear [62].

*Coxiella* was identified as part of the core microbiome (Fig 5). Rodents are recognized reservoirs of *Coxiella burnetii*, the causative agent of Q fever [63]. The detection of *Coxiella* DNA in wild capybaras highlights the importance of continued surveillance in wildlife populations, particularly in areas where contact with domestic animals occurs. Capybaras often inhabit ecotones—transitional zones between natural habitats and human-managed environments where they coexist with livestock and human populations. In Colombia, they are widely distributed across floodplains and savannas, where contact with domestic animals such as cattle, pigs, and poultry is common, creating ecological interfaces where microbial exchange may occur.

Considering the ecological flexibility and increasing presence of capybaras in anthropized landscapes, these findings support the inclusion of this species in future integrated One Health surveillance initiatives [64,65]. Capybaras are recognized amplifying hosts for *Rickettsia rickettsii* in Brazilian spotted fever–endemic areas, sustaining high tick densities and increasing human exposure risk in shared habitats [12,66]. The findings of this study highlight the importance of monitoring not only well-known zoonotic agents but also emerging or opportunistic microbes, which may silently circulate within wildlife populations. Future research should investigate their viability and pathogenicity, potential transmission routes whether through environmental exposure or vectors and their persistence in the environment, in order to better assess the risk of disease emergence at the human, animal and environment interface.

Capybaras’ interaction with both aquatic and terrestrial ecosystems also positions them as valuable bioindicators of environmental health [54]. Monitoring their microbiota can help track environmental microorganisms transmitted through soil, water, food, or intermediate hosts such as gastropods [67]. This has been observed in other rodents such as *Apodemus* spp. and *Myodes glareolus*, whose gut microbiota shifts in response to anthropogenic stressors like radiation [67]. In this study, genera such as *Blautia*, *Bacteroides*, *Prevotella*, and the *Rikenellaceae* RC9 gut group were not only differentially abundant across individuals (Fig 3) but consistently present in the core microbiome (Fig 5). These taxa are widely recognized as indicators of fecal contamination [68–70], highlighting their potential role in environmental surveillance.

This study has several limitations that should be acknowledged. First, the sample size per geographic location was limited, which may have reduced the statistical power to detect finer-scale differences in microbial composition across sites. Although patterns of variation were observed, particularly in fecal microbiota, larger and more balanced sampling across multiple regions would strengthen the generalizability of the findings. Second, the lack of detailed environmental data (e.g., vegetation type, water sources, human presence, or land use) limits the ability to identify ecological factors driving microbiome differences between populations. Third, the absence of epidemiological information from the sampled individuals, such as age, sex, diet, and health status, restricted the capacity to disentangle host-related factors influencing microbial communities. Future studies integrating microbiome, ecological, epidemiological, and spatial data will be essential to better understand the environmental and host-related determinants of microbial diversity in wild capybaras.

## Conclusion

Altogether, these findings provide baseline information on the microbiota composition of wild capybaras across multiple body sites. The identification of bacterial genera associated with fiber degradation is consistent with dietary adaptation in herbivorous rodents, while the detection of opportunistic or zoonotic-associated genera highlights the importance of continued surveillance at wildlife–human–livestock interfaces. These results should be interpreted descriptively due to the absence of individual host metadata such as age, sex, diet, and health status. Future studies integrating ecological, epidemiological, and host-level variables will be necessary to better understand microbiome drivers and functional implications in wild capybaras.

## Supporting information

S1 Table Number of reads obtained from 16S rRNA long-amplicon sequencing after quality control (trimming and filtering), for each biological sample type (saliva, feces, and blood) analyzed in the study. (XLSX)

S2 Table Venn diagram-based showing the distribution of bacterial genera shared and unique across saliva, blood, and feces samples. (XLSX)

S3 Table Code and scripts used for data processing and statistical analyses in this study. (DOCX)

S1 Fig Microbial community composition and diversity in saliva and fecal samples of capybaras. Panel A shows both the relative abundance of the 15 most frequent bacterial genera and their average abundance grouped by sampling location in saliva samples, while panel B presents the same information for fecal samples. Panel C displays alpha and beta diversity indices (Observed, Shannon, Inverse Simpson, and Chao1) for saliva, and panel D shows the corresponding diversity metrics for feces. Significance codes: *p < 0.05; **p < 0.01; ***p < 0.001. All visualizations were generated in RStudio using the ggplot2 package.
(TIFF)

S2 Fig Differential abundance analysis of bacterial genera in saliva samples from different sampling locations, performed with ANCOM-BC. (TIFF)
