# Supplementary material for: Exploring anatomical and geographical drivers of the microbiota in wild capybaras (Hydrochoerus hydrochaeris): Baseline Data for zoonotic risk assessment
Source: PLoS One. 2026 Mar 23;21(3):e0345409. doi: 10.1371/journal.pone.0345409 (PMC13008049; doi:10.1371/journal.pone.0345409)

**S3 Table. Code and scripts used for data processing and statistical analyses in this study.**

| **Analysis Category** | **Software / Package** | **Version** | **Script** | **Main Purpose** | **Key Parameters / Notes** | **Output** |
| --- | --- | --- | --- | --- | --- | --- |
| Data processing and microbiome object construction | R | v4.4.1 (2024-06-14) | microbiome_processing.R | Data import, formatting, and construction of microbiome objects | Default phyloseq workflows | Processed microbiome dataset |
| Taxonomic and compositional analysis | microbiome | 1.28.0 | taxonomic_analysis.R | Taxonomic aggregation and relative abundance calculations | Genus-level aggregation, compositional transformation | Relative abundance tables |
| Diversity analysis | phyloseq | 1.50.0 | diversity_analysis.R | Alpha and beta diversity estimation | Shannon, Simpson, Chao1; Bray–Curtis distance | Diversity metrics and distance matrices |
| Statistical testing | vegan | 2.7.1 | statistical_analysis.R | Group comparisons and community structure testing | Wilcoxon test, PERMANOVA (999 permutations), ANOSIM | Statistical test results |
| Ordination and visualization | ggplot2 | 3.5.2 | visualization.R | Ordination analyses and figure generation | NMDS / MDS ordination | Figures and ordination plots |

**Script Microbiome_processing.R**


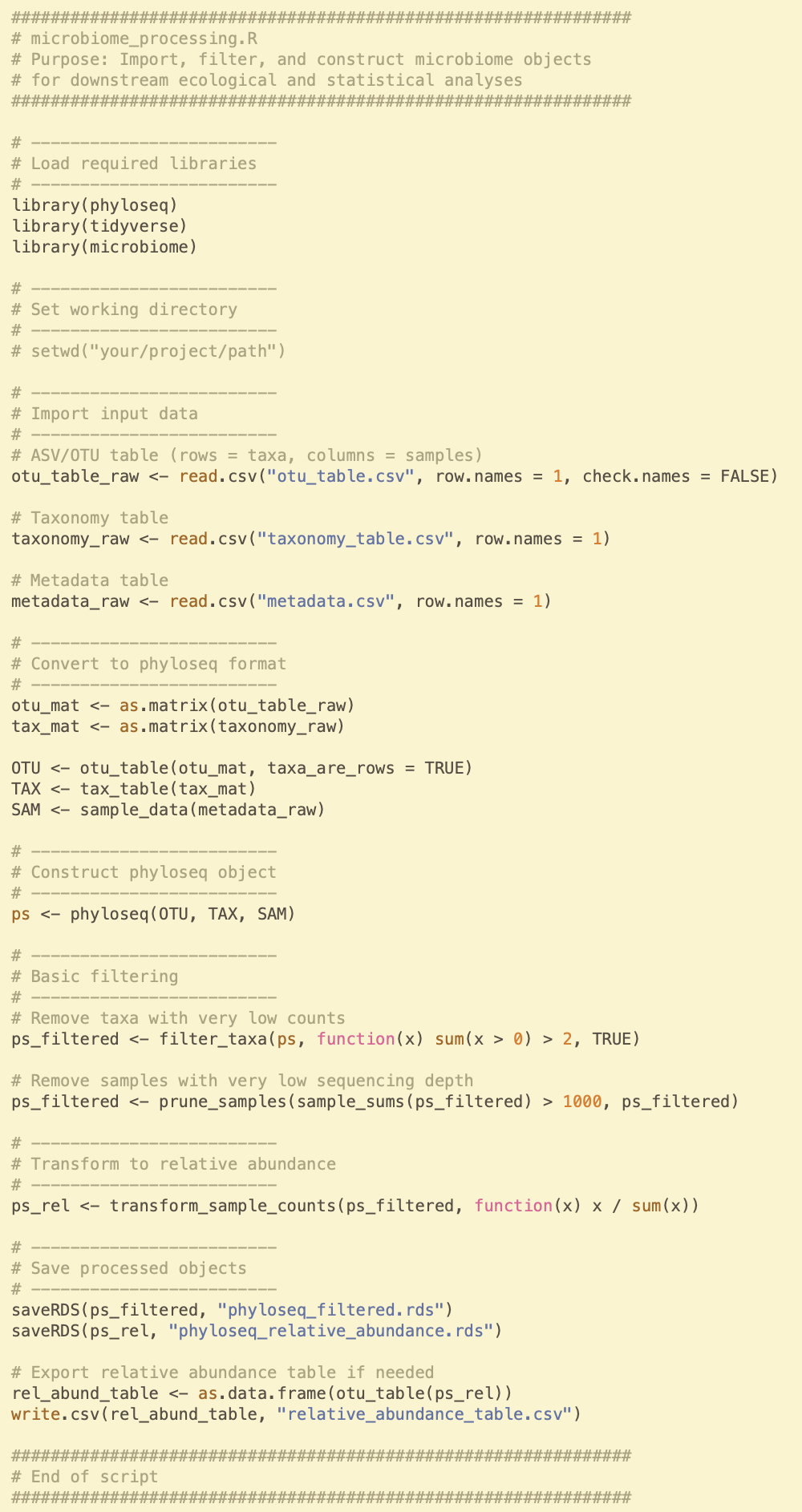


**Script** **Taxonomic_analysis.R**


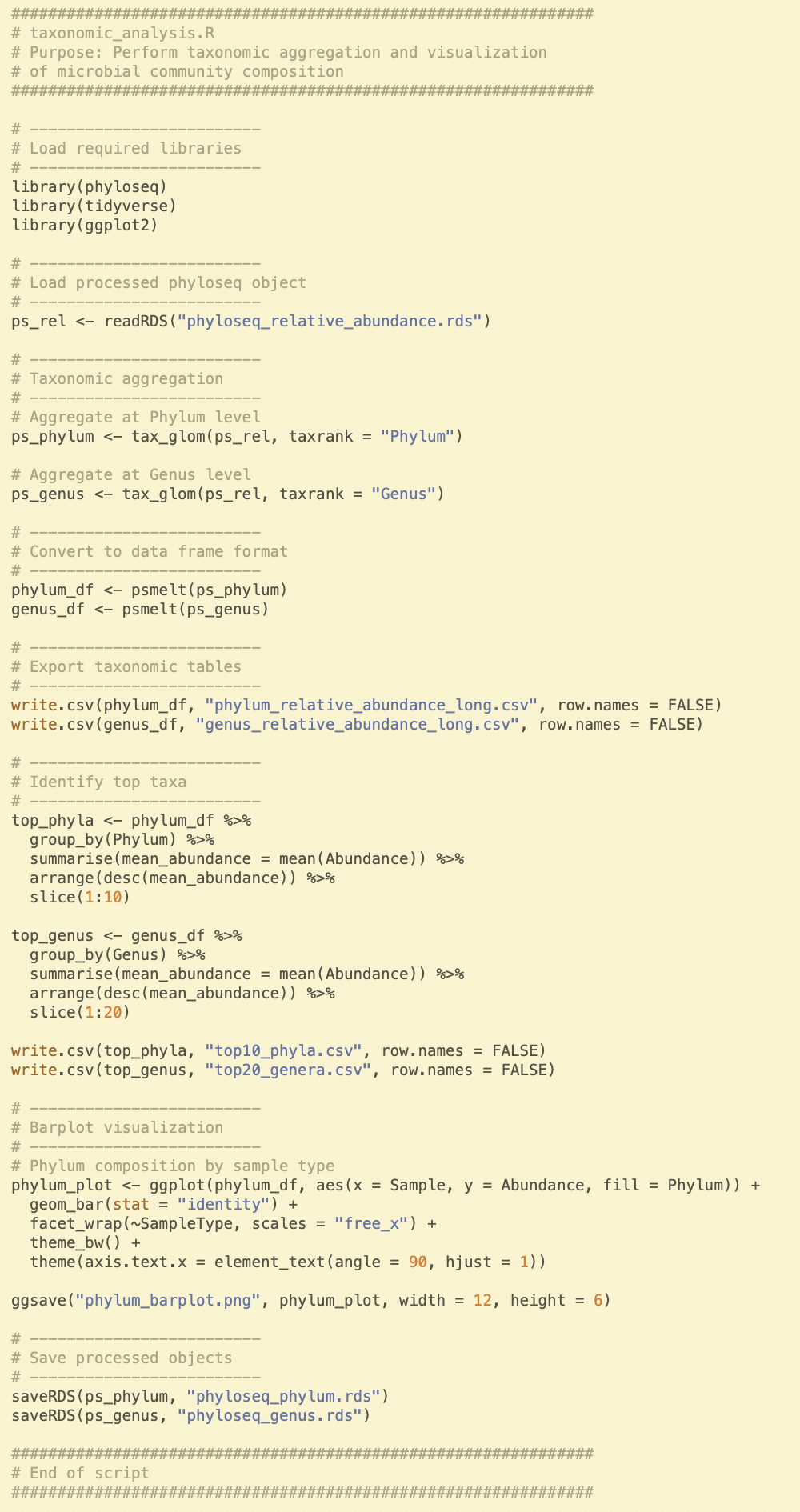


**Script** **Diversity_analysis.R**


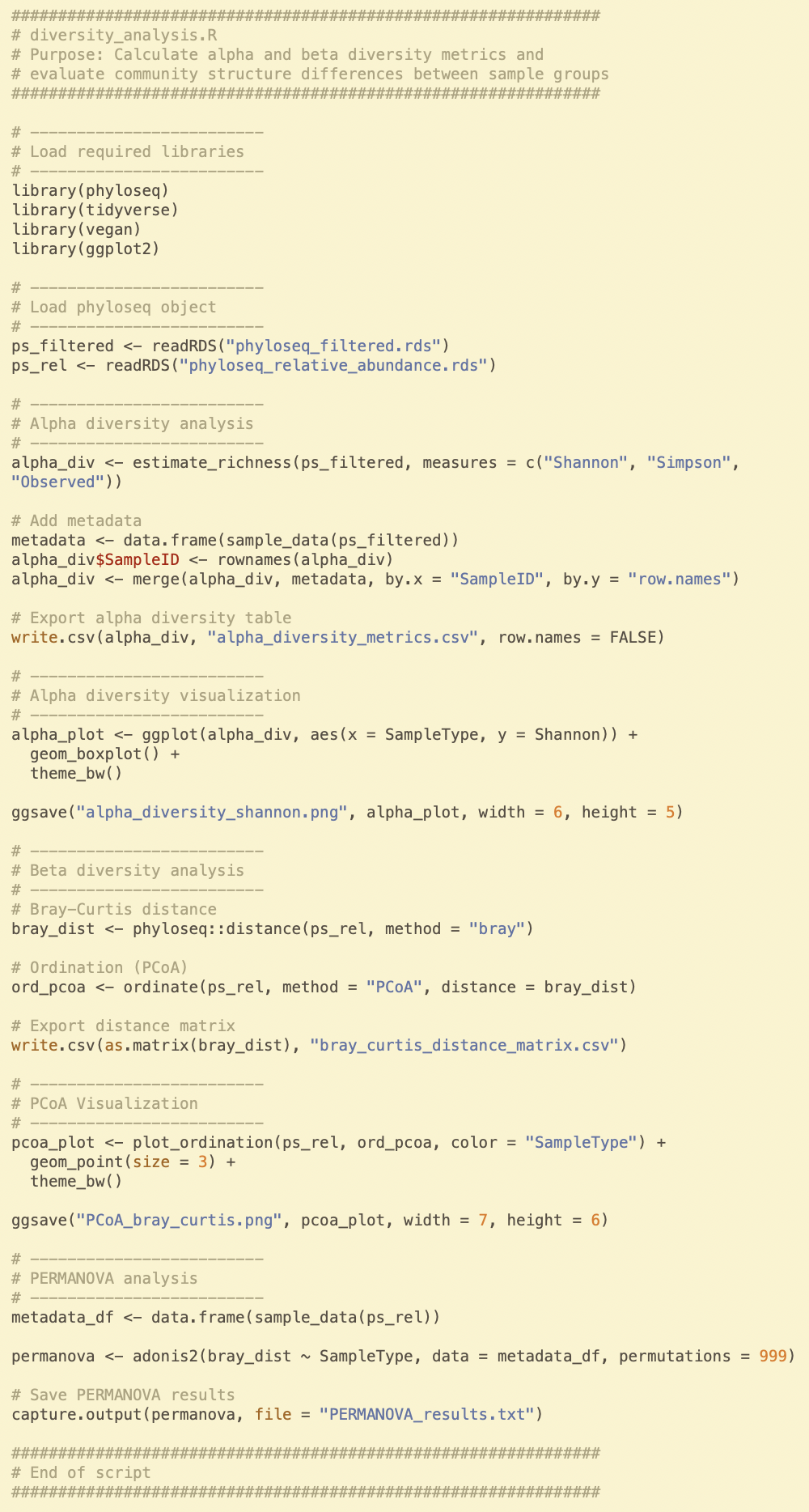


**Script** **Statistical_analysis.R**


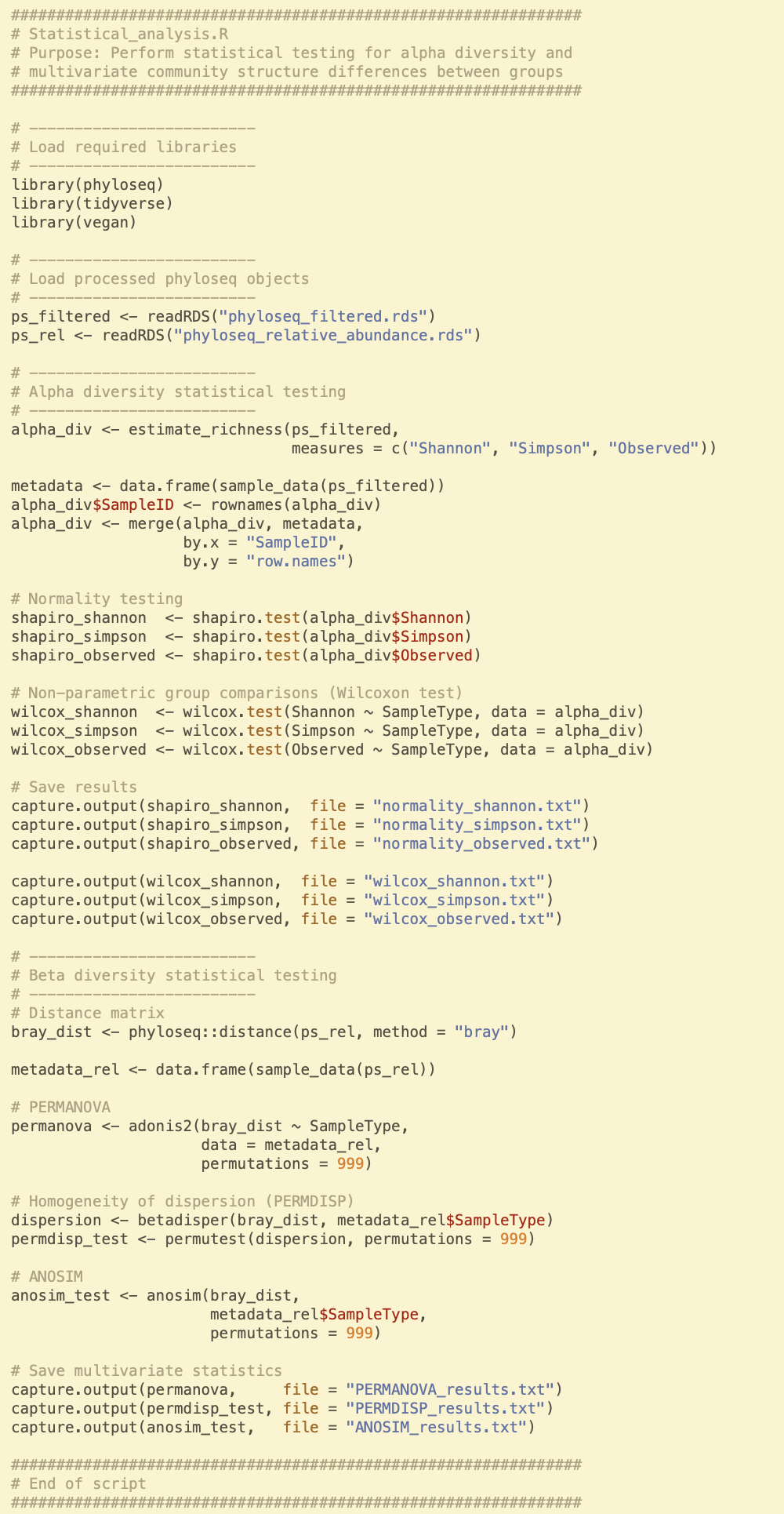


**Script** **Visualization.R**


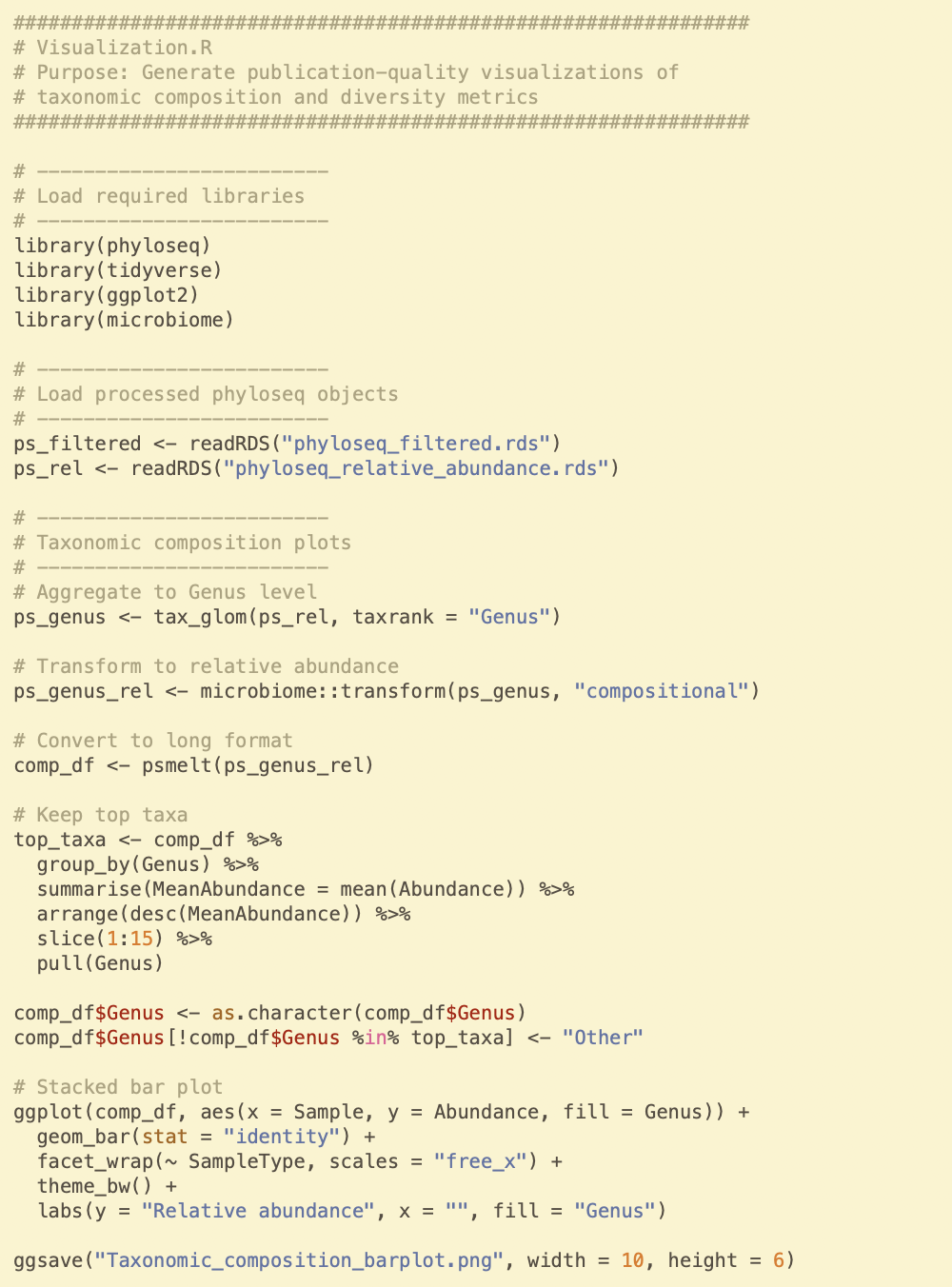


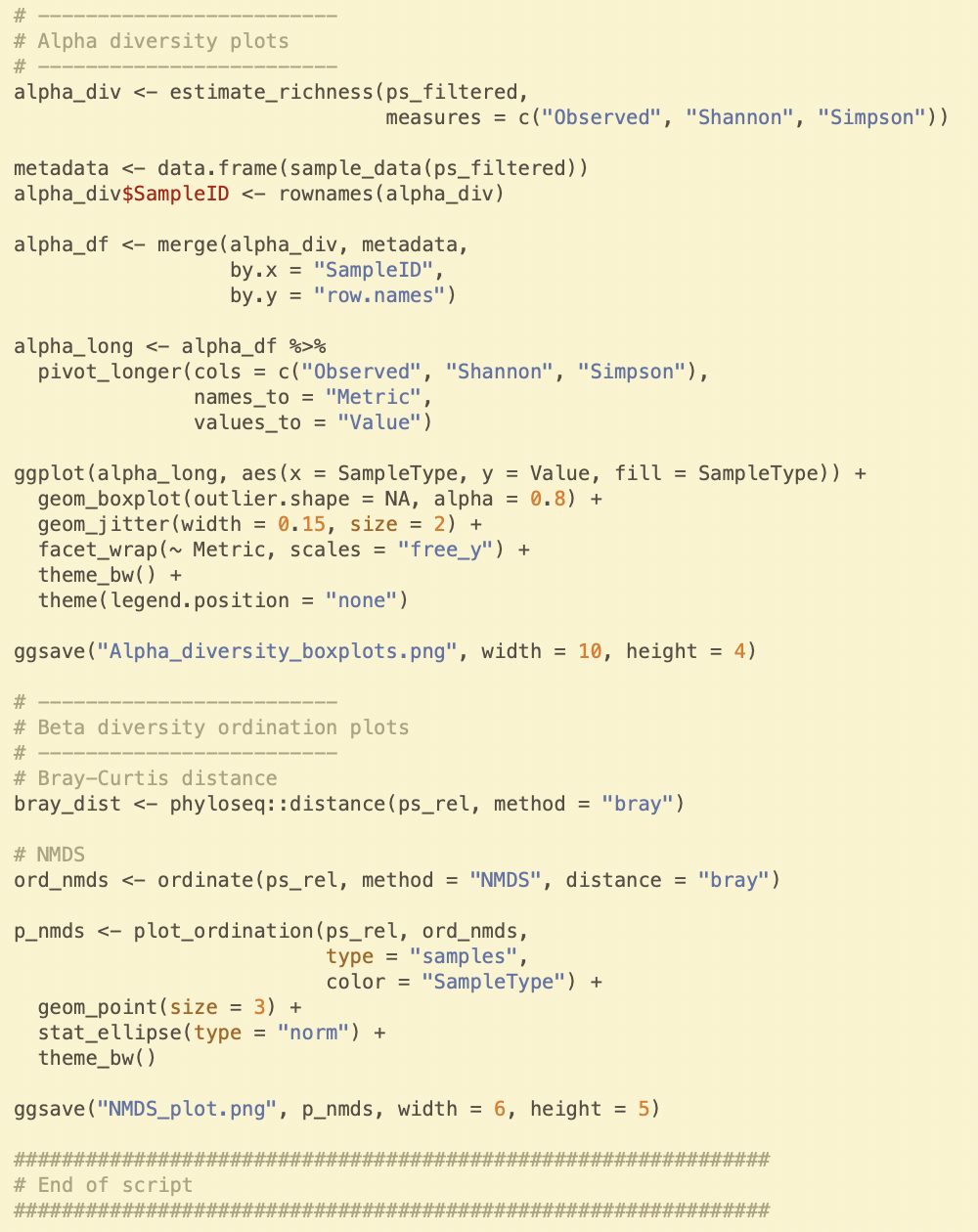

Supplement: S3 Table — (DOCX) [file pone.0345409.s003.docx]
