# Supplementary figures and images for: Exploring anatomical and geographical drivers of the microbiota in wild capybaras (Hydrochoerus hydrochaeris): Baseline Data for zoonotic risk assessment
Source: PLoS One. 2026 Mar 23;21(3):e0345409. doi: 10.1371/journal.pone.0345409 (PMC13008049; doi:10.1371/journal.pone.0345409)

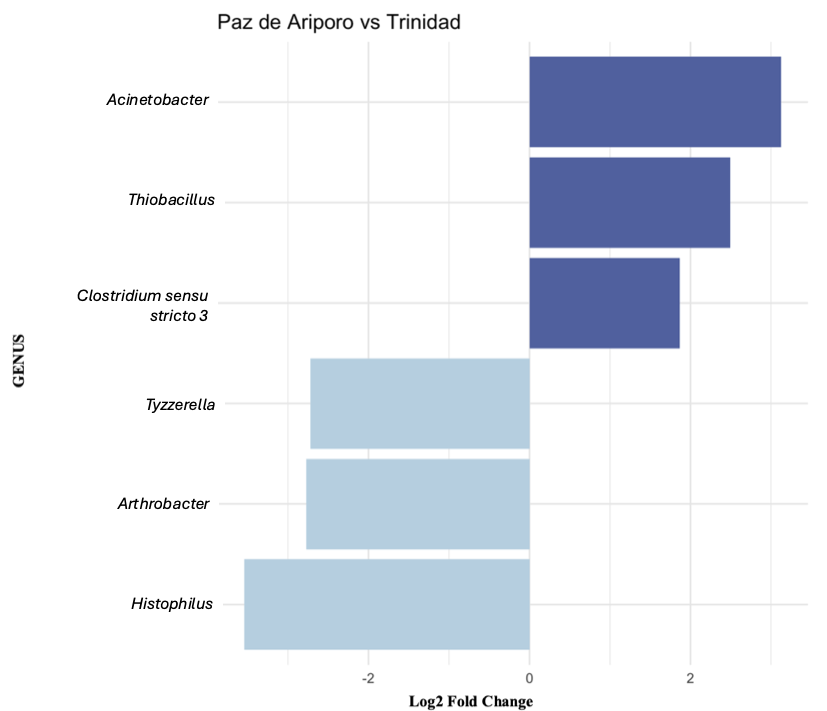

Supplement: S2 Fig — (TIFF) [file pone.0345409.s005.tiff]
